# Combinatorial Analysis of AT-Rich Interaction Domain 1A and CD47 in Gastric Cancer Patients Reveals Markers of Prognosis

**DOI:** 10.3389/fcell.2021.745120

**Published:** 2021-11-03

**Authors:** Qianfu Zhao, Qu Cai, Shanhe Yu, Jun Ji, Zhenggang Zhu, Chao Yan, Jun Zhang

**Affiliations:** ^1^Department of Oncology, Ruijin Hospital, Shanghai Jiao Tong University School of Medicine, Shanghai, China; ^2^State Key Laboratory of Medical Genomics, National Research Center for Translational Medicine at Shanghai, Shanghai Institute of Hematology, Ruijin Hospital Affiliated to Shanghai Jiao Tong University School of Medicine, Shanghai, China; ^3^Shanghai Key Laboratory of Gastric Neoplasms, Department of Surgery, Ruijin Hospital, Shanghai Institute of Digestive Surgery, Shanghai Jiao Tong University School of Medicine, Shanghai, China; ^4^State Key Laboratory of Oncogenes and Related Genes, Shanghai Jiao Tong University, Shanghai, China

**Keywords:** ARID1A, CD47, gastric cancer, prognosis, immunotherapy

## Abstract

**Background:** The AT-rich interaction domain 1A (*ARID1A*) is thought to be a tumor suppressive gene, and most of its mutations result in loss of expression of ARID1A protein. Combined with SIRPα on the surface of macrophages, CD47 on the surface of cancer cells can send an antiphagocytic “Don’t eat me” signal to the immune system that helps to avoid immune surveillance. However, the relationship between ARID1A and CD47 expression and their prognostic value in gastric cancer (GC) are still unknown.

**Methods:** In this study, we evaluated ARID1A and CD47 expression in 154 GC patients’ tissues using tissue microarray. Expressions of ARID1A and CD47 in GC cell lines were determined by western blot and quantitative reverse transcriptase–polymerase chain reaction (qRT-PCR) techniques, and cell membranous CD47 expression was quantified by flow cytometry. In addition, chromatin immunoprecipitation (ChIP)–qPCR was used to determine the aspects of regulation of *CD47* by ARID1A. The proportions of tumor-infiltrating immune cells were estimated on The Cancer Genome Atlas (TCGA) data set by using quanTIseq and EPIC algorithms. The infiltration of M1-polarized macrophages, M2-polarized macrophages, and regulatory T cells (Tregs) in GC tissues was determined by multispectral immunofluorescence.

**Results:** A significant correlation was found between loss of ARID1A and high expression of CD47 at protein level in GC. By integrating 375 bulk RNA sequencing samples from TCGA data set, we found that mutated *ARID1A* correlated with high *CD47* expression. In GC cell lines, knockdown of *ARID1A* significantly increased CD47 expression both at protein and mRNA levels as measured by western blot, qRT-PCR, and flow cytometry. Moreover, ChIP-qPCR revealed that *CD47* was a direct downstream target gene of ARID1A in GC. Utilizing univariate and multivariate survival analyses, we found that patients with ARID1A^loss^CD47^high^ expression had a worse prognosis. Estimation of infiltrating immune cells on TCGA data set showed that a higher infiltration proportion of M2 macrophages and Tregs was found in *ARID1A*^mutated^*CD47*^high^ expression subgroup. Furthermore, application of multispectral immunofluorescence revealed a higher infiltration proportion of M2 macrophages and Tregs in ARID1A^loss^CD47^high^ GC tissues.

**Conclusion:** Loss of ARID1A is strongly correlated with high CD47 expression in GC, and combination of ARID1A and CD47 is a promising prognosis factor in GC.

## Introduction

Gastric cancer (GC) is the third leading cause of cancer-related death worldwide with the sixth highest morbidity rate (Sung et al., 2021). Among cancers, GC remains to be a huge burden on the public health in China by showing the third highest incidence and mortality rate (Zhang et al., 2021). Because of the population growth and changes of the age structure, the incidence rate of GC is anticipated to be increasing in the future. Despite achievements in the development of diagnostic techniques, patients are often diagnosed at an advanced stage, thus resulting in a high mortality rate. Previous studies have shown that the occurrence and development of GC are closely related to the gene mutation and immune state (Nagarajan et al., 2012; Li et al., 2018; Lin et al., 2019).

The SWI/SNF complex was first identified as a protein complex important for cellular responses to mating-type switching (SWI) or sucrose fermentation (SNF) in the yeast (Lu and Allis, 2017). This complex uses the energy released by ATP hydrolysis to drive nucleosome movement and regulate structuring of chromatin (Roberts and Orkin, 2004). As a core component of SWI/SNF chromatin remodeling complexes, ARID1A participates in various biological processes, including gene transcription, DNA replication, and DNA damage repair (Shen et al., 2015). *ARID1A* is identified as a tumor suppressor gene and is mutated at a high frequency in diverse cancers (Wu and Roberts, 2013; Lo et al., 2021), such as ovarian clear cell carcinoma (∼57%), uterine corpus endometrial carcinoma (∼30%), and stomach adenocarcinoma (∼31%) (Lo et al., 2021). Recently, proof was provided for the context-dependent tumor-suppressive and oncogenic roles of *ARID1A* in the liver cancer (Sun et al., 2017). These findings prompt one to wonder if ARID1A plays a key role in the tumorigenesis and cancer development.

In recent years, immune checkpoint therapy has achieved a great success in cancer treatment and has shifted the paradigm in this field of research. Programmed death 1/programmed death 1 ligand (PD-1/PD-L1) and cytotoxic T lymphocyte–associated protein 4 (CTLA-4) are the most common target molecules for immune checkpoint therapy, and their targeting has shown clinical success in many solid tumors. However, the objective response rate of current immune checkpoint therapy is approximately only 20%, which is low (Topalian et al., 2012). Therefore, it is vital to find new immune checkpoints to develop new immune therapy drugs. CD47, also known as integrin-associated protein, is a cell membrane protein that belongs to the immunoglobulin superfamily (Vonderheide, 2015). CD47 is approximately 50 kDa, with an extracellular N-terminal IgV domain, five transmembrane domains, and a short intracytoplasmic C-terminal domain. CD47 is overexpressed in almost all tumor cells and closely related to clinical prognosis. Besides, in combination with SIRPα on the surface of macrophages, CD47 can send an antiphagocytic “Don’t eat me” signal to the immune system that helps cancer cells avoid immune surveillance (Morrissey et al., 2020). Thus, CD47 is expected to become an emerging targeted immune checkpoint.

Results of the clinical and non-clinical research accumulated over the years indicate that ARID1A deficiency is closely related to the immune checkpoint therapy, such as anti–PD-L1 therapy (Shen et al., 2018). However, the relationship between ARID1A and CD47 in GC has rarely been the focus of a study. In the present study, we mainly focused on examining the expression levels of ARID1A and CD47 and their expression relationship and elucidating their prognostic value in GC patients.

## Materials and Methods

### Patients and Samples

Tissue microarray used in this research included a total of 159 GC samples that were purchased from Shanghai Outdo Biotech Co., Ltd. The samples were collected between June 2011 and May 2013, and the follow-up ended on June 2017. After ruling out samples with missing data (3 samples) and distant metastasis (2 samples), 154 samples were included in the present study. Patients received neither chemotherapy nor radiation therapy before surgery. All the samples were validated as gastric adenocarcinoma by the researchers. Clinicopathologic parameters, including sex, age, tumor location, tumor histological grade, tumor size, T stage, N stage, tumor-node-metastasis (TNM) stage, and human epidermal growth factor receptor 2 (HER2) status, were collected. The tumor stage was identified using the eighth version of the TNM staging system of the American Joint Committee on Cancer. The overall survival (OS) time was defined from postoperative to death or to the end of the follow-up. Written informed consents were provided by Shanghai Outdo Biotech Co., Ltd., and the research was conducted in accordance with the recognized ethical guidelines. The studies that involved human participants were reviewed and approved by the Ethics Committee of Shanghai Jiao Tong University School of Medicine Affiliated Ruijin Hospital. The patients provided written informed consent to participate in this study.

### Immunochemistry Staining and Evaluation

Tissue microarray was established, and immunochemistry was conducted following the EnVision two-step procedure of Dako REAL^TM^ Envision^TM^ Detection System (Dako, Agilent Technologies, CA, United States) (Ji et al., 2019). In brief, the sections were deparaffinized and rehydrated before being heated in a microwave oven for antigen retrieval. After having been washed with phosphate-buffered saline (PBS) three times, the sections were incubated with 3% hydrogen peroxide to block the endogenous peroxidase activity. Afterward, the sections were incubated with 1 × Animal-Free Blocking Solution (Cell Signaling Technology, United States) for 1 h at room temperature to reduce non-specific staining. After blocking, the sections were incubated with the primary antibodies overnight at 4°C. On the next day, they were incubated with the horseradish peroxidase (HRP)–labeled second antibodies at 37°C for 30 min and then were incubated with diaminobenzidine solution for visualization.

**FIGURE 1:**
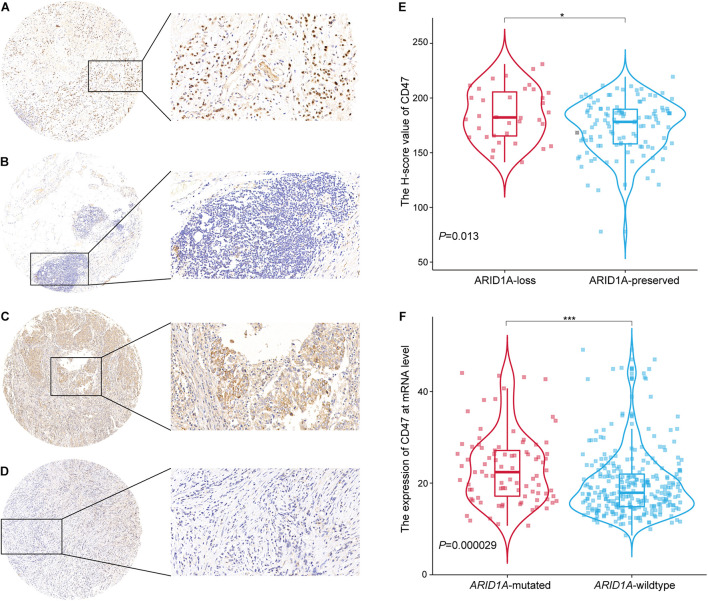
Expression of ARID1A and CD47 and their relationship in GC tissues. **(A,B)** Representative IHC images of ARID1A staining, ARID1A-preserved expression **(A)**, ARID1A-loss expression **(B)**. **(C,D)** Representative IHC images of CD47 staining, CD47 high expression **(C)**, CD47 low expression **(D)**. **(E)** Relationship between loss/preserved expression of ARID1A and CD47 expression (*n* = 154). **(F)** Relationship between mutated/wild-type expression of *ARD1A* and *CD47* expression (data from TCGA, *n* = 375). *p < 0.05, ***p < 0.001.

Anti-ARID1A mouse monoclonal antibody (sc-32761; 1:50 dilution; Santa Cruz Biotechnology, United States) was used to detect the expression level of ARID1A protein in GC tissues. ARID1A was located in the cellular nuclear area and was dyed yellow to varying degrees in cancer cells. Using CaseViewer (magnification, × 40; 3DHISTECH Ltd.), the expression status of ARID1A protein was determined by two independent investigators. Loss of ARID1A was identified once the nuclear staining was vacant in more than 10% of cancer cells, and the rest of the cases were defined as “preserved” (Kim et al., 2016; Figures 1A,B).

Anti-CD47 rabbit polyclonal antibody (20305-1-AP; 1:50 dilution; Proteintech Group, United States) was applied to identify CD47 expression level in tissues. The expression of CD47 in GC tissues was evaluated by histochemistry score (*H* score). *H* score = Σ(pi × *i*) = (percentage of weak intensity area × 1) + (percentage of moderate intensity area × 2) + (percentage of strong intensity area × 3), where *i* is the staining intensity, including negative without staining (*i* = 0), weak positive (*i* = 1), medium positive (*i* = 2), and strong positive (*i* = 3), and pi is proportion of the positive cells for the corresponding staining intensity (Paschalis et al., 2019). The median of *H* score was defined as the threshold value to distinguish low and high CD47 expression group. The cutoff value is 179.86 (Figures 1C,D).

### Cell Lines and Cell Culture

GC cell lines (BGC-823, MKN-28, SNU-1, SUN-5, SNU-16, MKN-45, KATO-III, BGC-803, AGS, SGC-7901, and NCI-N87) and GSE-1 cell lines were preserved in the Ruijin Hospital (Shanghai, China) (Wang et al., 2019). Cells were cultured in DMEM (Dulbecco modified eagle medium) or RPMI-1640 medium supplemented with 10% fetal calf serum at 37°C and with 5% CO_2_.

### Lentivirus Transduction

The ARID1A lentiviral short hairpin RNAs (shRNAs) were supplied by GenePharma (Shanghai, China). The target sequences are listed in Supplementary Table 1. The shRNAs were cloned into PGMLV-SB3 (PGMLV-hU6-MCS-CMV-Puro-WPRE) vector, and an empty vector was used as the control vector. For lentivirus transduction, cells were transduced following the manufacturer’s instruction and were selected by puromycin (cat. Ant-pr-5; Invivogen) with the concentration of 2 μg/mL. The expression of ARID1A in BGC-823 and MKN-28 was confirmed by western blot and quantitative reverse transcriptase–polymerase chain reaction (qRT-PCR).

### Western Blot Analysis

Proteins were fractionated by 10% sodium dodecyl sulfate–polyacrylamide gel electrophoresis gels and were transferred to polyvinylidene fluoride (PVDF) membranes. The primary antibodies included ARID1A (12354S; 1:1,000 dilution; Cell Signaling Technology, United States), CD47 (20305-1-AP; 1:1,000 dilution; Proteintech Group, United States), and GAPDH (51332S; 1:1,000, Cell Signaling Technology, United States). PVDF membranes were blocked by 5% skim milk for 2 h at room temperature and then were incubated with primary antibodies overnight at 4°C. The membranes were then incubated with HRP-conjugated secondary antibodies (7074, 7076; 1:2,500, Cell Signaling Technology, United States). ECL western blotting substrate (cat. P10300, NCM Biotech, China) and infrared imaging system (LI-COR Biosciences) were used to visualize the protein bands.

### Real-Time Quantitative Reverse Transcriptase–Polymerase Chain Reaction

RNA was extracted by Trizol reagent method. Reverse transcription in 20-μL system was performed following the protocol of Fasting gDNA Dispelling RT SuperMix kit (KR118-03; TIANGEN, China). Primers for qRT-PCR were *ARID1A*: 5′-GCATCCTTCCATGAACCAAT-3′ (forward), 5′-CCCATGCCTGTGTGTATCTG-3′(reverse);*GAPDH*:5′-GGAC CTGACCTGCCGTCTAG-3′ (forward), 5′-GTAGCCCAGGA TGCCCTTGA-3′(reverse);*CD47*:5′-AGAAGGTGAAACGATC ATCGAGC-3′ (forward), 5′-CTCATCCATACCACCGGATCT-3′ (reverse). Relative mRNA expression was normalized to *GAPDH* and calculated by 2^–△CT^ method. Each experiment was performed in triplicate.

### Flow Cytometry

Surface expression of CD47 in GC cells was measured by flow cytometry. In brief, equal amounts of cells were harvested, were Fc-blocked, and were incubated with anti-CD47 antibody (PE) (cat. 12283-MM07-P; SinoBiological) or isotype control for 30 min at room temperature. Afterward, the cells were washed three times and were resuspended in 1% fetal bovine serum/PBS. The fluorescence intensity of CD47 in GC cells was collected on a BD Calibur flow cytometer and was analyzed by FlowJo V10 software.

### Chromatin Immunoprecipitation Assay

Chromatin immunoprecipitation (ChIP) assays were performed as previously described (Yu et al., 2018). Briefly, BGC-823 and MKN28 cells were harvested and crosslinked in 1% formaldehyde at room temperature, and the fixation reactions were quenched by adding glycine to a final concentration of 0.125 M. After sonication, we separately incubated the soluble chromatins with the antibodies anti-ARID1A (12354S, Cell Signaling Technology) or control IgG (2729, Cell Signaling Technology). Afterward, chromatin immunocomplexes were precipitated with protein A (Millipore, 16-661). The immunoprecipitated complex was washed, and DNA was extracted and purified by QIAquick PCR Purification Kit (Qiagen). ChIP DNA was analyzed by qPCR using specific primers, and the data were normalized by input DNA. The primers for ChIP-qPCR are the following: human *CD47* (5′-AAAGAAGGGGATCCCTAGCA-3′, 5′-CCATCTCCAAATGCACACAC-3′).

### Public Database Analysis

Public data from The Cancer Genome Atlas (TCGA), including that of 375 GC patients was included in our analysis. Other cancer data from TCGA including colon adenocarcinoma (COAD), lung adenocarcinoma (LUAD), breast invasive carcinoma (BRCA) and kidney renal clear cell carcinoma (KIRC) was also included in this analysis. TCGA data were downloaded from the Genomic Data Commons data portal,^1^ and *ARID1A* mutation information was acquired from the cBioPortal.^2^ The cutoff value of *CD47* in TCGA data sets was determined as median. Tumor-infiltrating immune cells were estimated on the TCGA data by quanTIseq^3^ (Finotello et al., 2019) and EPIC^4^ (Racle et al., 2017).

### Multispectral Immunofluorescence

Multispectral immunofluorescence staining was performed following the manufacturer’s instructions of Opal 4-Color Manual IHC Kit (NEL794001KT; PerkinElmer, Waltham, MA, United States). The Opal kit uses tyramine signal amplification conjugated fluorophores to detect targets within an immunofluorescence assay. Initially, the slides were baked in the oven at 65°C for 1 h, dewaxed with xylene, and rehydrated through a graded series of ethanol solutions. After rehydration, slides were fixed in 10% neutral buffered formalin for 20 min. The slides were then placed in microwave for 45 s at 100% power followed by additional 15 min at 20% power in AR buffer. The slides were blocked with blocking buffer (PerkinElmer) and incubated in a humidified chamber for 10 min at room temperature. After removal of the blocking buffer, the slides were incubated with primary antibodies overnight at 4°C. Primary antibodies including CD11c (Abcam, ab11029, 1:75), CD163 (Abcam, ab182422, 1:800), and Foxp3 (Abclonal, A12051, 1:50). The slides were sequentially incubated with Opal Polymer HRP MS + Rb (PerkinElmer) and Opal Fluorophore Working Solution (pal 520, Opal 570, Opal 670; 1:50 dilution) for 10 min at room temperature, respectively. Finally, the slides were incubated with DAPI solution (PerkinElmer) for 5 min at room temperature and were mounted with ProLong Diamond Antifade Mountant (Invitrogen). Imaging was performed using Vectra Quantitative Pathology Imaging Systems (PerkinElmer), and image analysis was performed using the InForm Advanced Image Analysis software (inForm 3.0; PerkinElmer).

### Statistical Analysis

The data were analyzed using GraphPad Prism (version 8.0.1), statistical product and service solution (SPSS) (version 22), R (version 4.1.0), and Rstudio (version 4.0.4) software. A *χ*^2^-test was used to analyze the relationship between clinicopathologic parameters and ARID1A status and CD47 expression level. Survival analysis was performed using the Kaplan–Meier method, log-rank test, and Cox regression analysis under various conditions. Analysis of variance and *post hoc* test were used to calculate the difference in the means of multiple groups. Non-parametric test was used to analyze the data, which did not follow normal distribution. *p* < 0.05 was considered statistically significant.

## Results

### Clinicopathological Characteristics of 154 Gastric Cancer Patients

The clinicopathological characteristics of 154 GC patients enrolled in this study are shown in Table 1. In the registered patients, 66.2% of patients were males, and 33.8% were females. In line with a previous study, the incidence rate ratio of male to female was approximately 2:1 in China (Sung et al., 2021). Most patients were 60 years or older (119 cases, 77.3%). For the histological grading, the samples of 66.2% of the patients were poorly differentiated. Regarding T stage, we found that most of the patients were at T4 stage, indicative of the fact that most of the patients were diagnosed at an advanced stage. For the classification according to HER2 status, 116 of patients (75.3%) were found to be negative/weak positive (1 +), which assigned them as HER2 negative, and 9.1% of them were strong positive (3 +), which assigned them as HER2 positive.

**TABLE 1:** Clinicopathological characteristics of 154 GC patients.

Factors	No. of patients	Percent
**Age (years)**		
<60	35	22.7
≥60	119	77.3
**Gender**		
Male	102	66.2
Female	52	33.8
**Location**		
Proximal	14	9.1
Middle	70	45.5
Distal	70	45.5
**Histological grade**		
Moderate	52	33.8
Poor	102	66.2
**Tumor size (cm)**		
<5	81	52.6
≥5	73	47.4
**N stage**		
N0	44	28.6
N1	33	21.4
N2	34	22.1
N3	43	27.9
**T stage**		
T1	4	2.6
T2	15	9.7
T3	18	11.7
T4	117	80
**TNM stage**		
I	15	9.7
II	36	23.4
III	103	66.9
**HER2 status**		
0/1+	116	75.3
2+	24	15.6
3+	14	9.1

### A Strong Correlation Exists Between a High Expression Level of CD47 and Loss/Mutation of AT-Rich Interaction Domain 1A in Gastric Cancer

Immunohistochemical (IHC) staining was carried out on GC tissue microarrays to evaluate ARID1A (Figures 1A,B) and CD47 expression (Figures 1C,D). According to the evaluation criteria, loss of ARID1A expression was identified in 36 cases of GC tissues (23.4%). The remaining 118 cases (76.6%) exhibited preserved expression of ARID1A. Regarding CD47 expression, 68 of GC tissues (44.2%) were identified as low expression tissues, and 86 of them (55.8%) were identified as high-expression ones. Next, we investigated the correlation between the expression of ARID1A and CD47 in GC tissues. More specifically, in GC tissues, the *H* score of CD47 was higher in the ARID1A-loss group than in the ARID1A-preserved group (Figure 1E). Previous studies have reported that most *ARID1A* mutations are inactivated non-sense mutations or frameshift mutations, which result in the loss of ARID1A protein expression (Wang et al., 2004; Wiegand et al., 2010; Wu et al., 2014). Thus, we examined the *ARID1A*-mutated expression as a surrogate of ARID1A-loss expression in TCGA data set. An analogous trend was also identified in CD47 expression at transcriptional level through analysis of the data from the TCGA. More specifically, the expression of CD47 in *ARID1A*-mutated group was found to be higher than that of the *ARID1A* wild-type group (Figure 1F). Furthermore, we found that the mRNA expression level of *CD47* was higher in *ARID1A*-mutated samples than *ARID1A* wild-type samples in COAD, LUAD, BRCA, and KIRC, respectively (Supplementary Figure 1). Putting side-by-side the information obtained so far, we concluded that loss of ARID1A was significantly correlated with high CD47 expression in GC.

### AT-Rich Interaction Domain 1A Status and CD47 Expression Associate With Clinicopathological Features of Gastric Cancer

The relationships between ARID1A status, CD47 expression, and clinicopathologic features of GC patients are shown in Table 2. As shown in Table 2, ARID1A status and CD47 expression level were significantly associated with T stage. Notably, in early T stage (T1 and T2) cases, the expression of ARID1A was always preserved, and in the majority of instances, the expression of CD47 was low. These findings in early T stage samples might be explained by the tumor suppressor effect of ARID1A and the lack of CD47-mediated antiphagocytosis effect (Wu and Roberts, 2013; Morrissey et al., 2020). As for TNM stage, the proportion of ARID1A-loss cases increased with the progression of the TNM stage, and the proportion of high CD47 cases appeared to show a similar trend. These might indicate that ARID1A is often lost at the advanced stages of GC. Moreover, we also found that ARID1A status was correlated with tumor size. No significance difference in the expression of ARID1A was found between groups with age, gender, tumor location, histological grade, and N stage. Besides, no significant difference in the expression of CD47 was found between groups with age, gender, tumor location, histological grade, tumor size, N stage, and TNM stage.

**TABLE 2:** The correlation between ARID1A status and CD47 expression and clinicopathological characteristics.

		ARID1A expression			CD47 expression		
			
	Total (*n* = 154)	Loss (%) (*n* = 36)	Preserved (%) (*n* = 118)	*P*-value	Low (%) (*n* = 68)	High (%) (*n* = 86)	*P*-value
**Age (years)**				0.591			0.17
<60	35	7(20.0)	28(80.0)		19(54.3)	16(45.7)	
≥60	119	29(24.4)	90(75.6)		49(41.2)	70(58.8)	
**Gender**				0.094			0.484
Male	102	28(27.5)	74(72.5)		43(42.2)	59(57.8)	
Female	52	8(15.4)	44(84.6)		25(48.1)	27(51.9)	
**Location**				0.327			0.351
Proximal	14	5(35.7)	9(64.3)		8(57.1)	6(42.9)	
Middle	70	13(18.6)	57(81.4)		33(47.1)	37(52.9)	
Distal	70	18(25.7)	52(74.3)		27(38.6)	43(61.4)	
**Histological grade**				0.642			0.989
Moderate	52	11(21.2)	41(78.8)		23(44.2)	29(55.8)	
Poor	102	25(24.5)	77(75.5)		45(44.1)	57(55.9)	
**Tumor size (cm)**				**0.002**			0.089
<5	81	11(13.6)	70(86.4)		41(50.6)	40(49.4)	
≥5	73	25(34.2)	48(65.8)		27(37.0)	46(63.0)	
**N stage**				0.317			0.091
N0	44	6(13.6)	38(86.4)		22(50.0)	22(50.0)	
N1	33	8(24.2)	25(75.8)		11(33.3)	22(66.7)	
N2	34	10(29.4)	24(70.6)		11(32.4)	23(67.6)	
N3	43	12(27.9)	31(72.1)		24(55.8)	19(44.2)	
**T stage**				**0.011**			**0.024**
T1	4	0(0.0)	4(100.0)		4(100.0)	0(0.0)	
T2	15	0(0.0)	15(100.0)		9(60.0)	6(40.0)	
T3	18	4(22.2)	14(77.8)		9(50.0)	9(50.0)	
T4	117	32(27.4)	85(72.6)		46(39.3)	71(60.7)	
**TNM stage**				**0.008**	46(39.4)		0.054
I	15	0(0.0)	15(100.0)		11(73.3)	4(26.7)	
II	36	7(19.4)	29(80.6)		14(38.9)	22(61.1)	
III	103	29(28.2)	74(71.8)		43(41.7)	60(58.2)	

**P*-value < 0.05 is displayed in bold.*

### AT-Rich Interaction Domain 1A Regulates CD47 Expression Levels in Gastric Cancer Cell Lines

**FIGURE 2:**
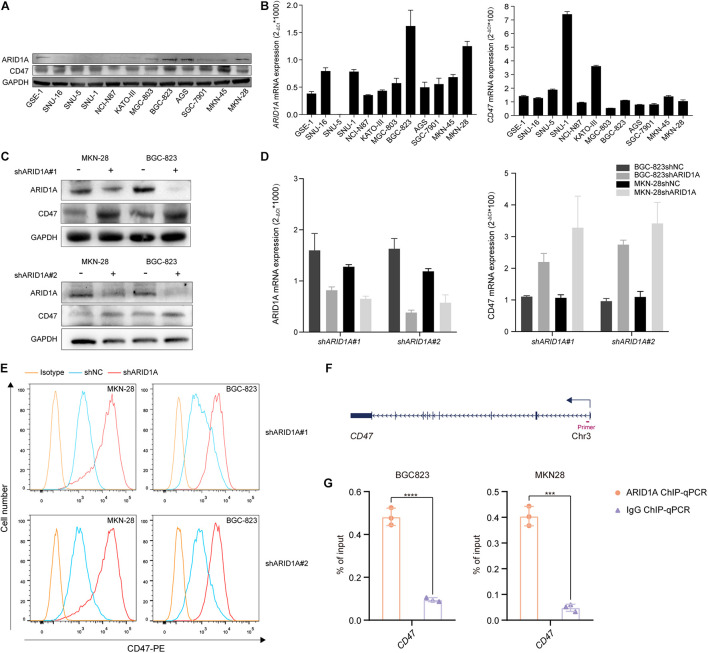
Loss of ARID1A increases CD47 expression. **(A,B)** Protein and mRNA expression levels of ARID1A and CD47 among various GC cell lines. **(C–E)** Knockdown of ARID1A significantly increased total and membranous CD47 protein levels, and *CD47* mRNA level measured by western blot, flow cytometry and qRT-PCR in GC cell lines. **(F)** Schematic illustration of the relative positions of qPCR probes to putative *CD47* promoter region for ChIP-qPCR experiments. **(G)** ARID1A binding to the promoter region of CD47 was determined by ChIP-qPCR in BGC-823 and MKN28 cells, respectively. ***p < 0.001, ****p < 0.0001.

To validate the relationship between ARID1A and CD47, we examined the expression of ARID1A in GC cell lines using western blot and qRT-PCR (Figures 2A,B). We found that the expression of ARID1A was depleted in SNU-16, SNU-5, and SNU-1, and these cell lines were poorly differentiated. Cell lines MKN-28 and AGS were more differentiated, and the expression of ARID1A was relatively higher in them. To study the loss of ARID1A in GC cell lines, we chose BGC-823 and MKN28 cells for their relative abundant expression and found that CD47 expression level was increased at the protein and mRNA level after *ARID1A* knockdown (Figures 2C,D). Besides, flow cytometry analysis showed that the expression of membranous CD47 was also significantly increased after *ARID1A* knockdown (Figure 2E). Analogous findings were observed using another shRNA. Notably, the extent of the correlation between expressions of ARID1A and CD47 in GC cell lines was the same as that observed in GC tissues, which is indicative of a strong association between loss of ARID1A and a high expression of CD47. To investigate how ARID1A regulates CD47 expression in GC, ChIP-qPCR assay was performed in BGC-823 and MKN28 cell lines, and the results revealed that *CD47* was a direct downstream target gene of ARID1A in GC (Figures 2F,G).

### ARID1A^loss^CD47^high^ Expression Indicates Poor Prognosis in Gastric Cancer Patients

**FIGURE 3:**
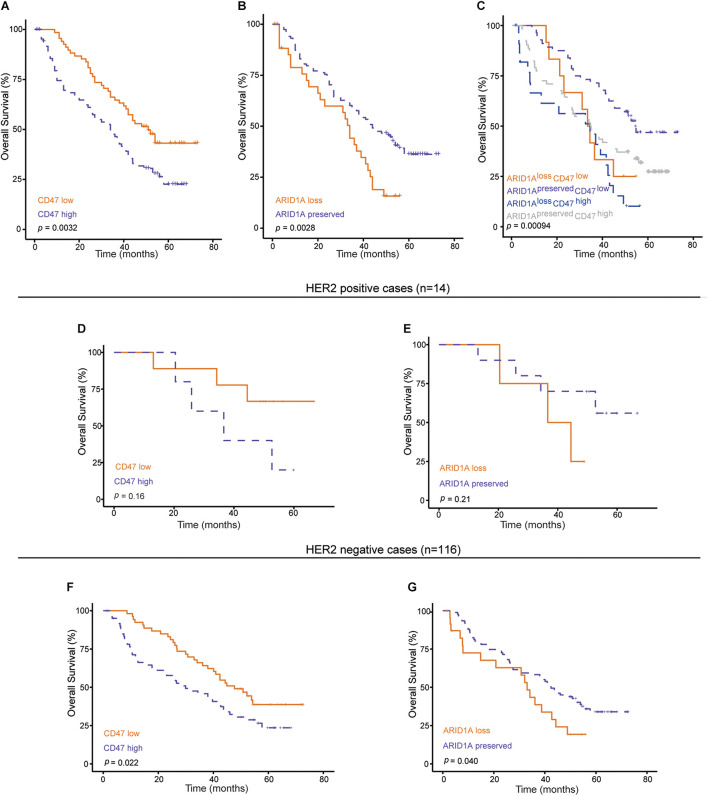
Kaplan–Meier curves under various conditions. **(A,B)** Survival curves according to ARID1A status or CD47 expression (*n* = 154). **(C)** Survival curves according to ARID1A status combined with CD47 expression level (*n* = 154). **(D–G)** Survival curves according to ARID1A status or CD47 expression with HER2 positive or negative.

To analyze the prognostic value of ARID1A and CD47 in GC, Kaplan–Meier survival analysis and log-rank test were conducted. The endpoint of survival analysis was OS time. As shown in Figure 3A, the low expression of CD47 was correlated with a better outcome for GC patients. Meanwhile, loss of expression of ARID1A was associated with a poorer outcome in such patients (Figure 3B). The mutation of tumor suppressor gene and a change in the immune microenvironment contribute, to a large extent, to tumor progression. Thus, we combined ARID1A status with CD47 expression to investigate the impact on OS of GC patients. Among all cases, cases in the ARID1A^preserved^CD47^low^ expression subgroup had the longest OS time (median OS = 53.9 months) than any other subgroups, whereas the outcome of cases in ARID1A^loss^CD47^high^ expression subgroup was the worst (median OS = 20.4 moths) (Figure 3C). It has been found that HER2 is overexpressed in approximately 20% of GC patients, and anti-HER2 targeted therapy significantly improves the prognosis of HER2-positive GC patients (Oh and Bang, 2020). To evaluate the therapeutic value of ARID1A and CD47, HER2 positivity–stratified analysis was conducted. In HER2-negative cases, loss of ARID1A expression or high CD47 expression indicated a poor outcome (Figures 3F,G). However, analysis did not reveal a statistically significant difference in the outcome of HER2-positive cases (Figures 3D,E). Thus, these results indicate that GC patients with ARID1A^preserved^CD47^low^ might have a better clinical outcome.

**FIGURE 4:**
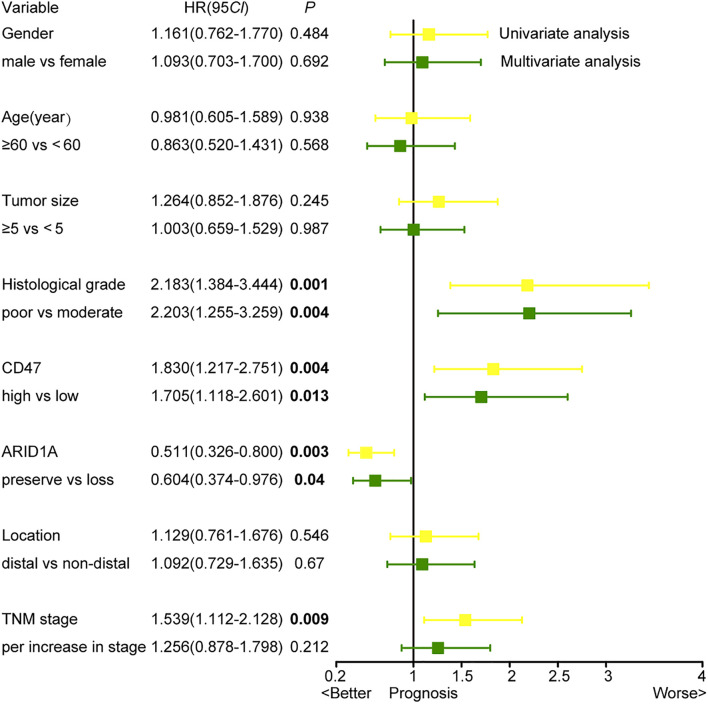
Univariate and multivariate analyses based on clinicopathological characteristics.

Cox regression analysis was performed to find out independent prognostic factors for the OS of GC. Factors including gender, age, tumor size, histological grade, tumor location, TNM stage, ARID1A status, and CD47 expression were taken into multivariate survival analysis. The results showed that CD47 [multivariate analysis: hazard ratio (HR) = 1.705; 95% confidence interval (CI) = 1.118–2.601; *p* < 0.01] and ARID1A (multivariate analysis: HR = 0.604; 95% CI = 0.374–0.976; *p* < 0.05) were independent prognostic factors for the OS of GC patients (Figure 4). In addition, histological grade was also identified as an independent prognostic factor for GC patients.

### Lower Infiltration of M2 Macrophage and Regulatory T Cells Was Found in ARID1A^preserved^CD47^low^ Gastric Cancer Patients

**FIGURE 5:**
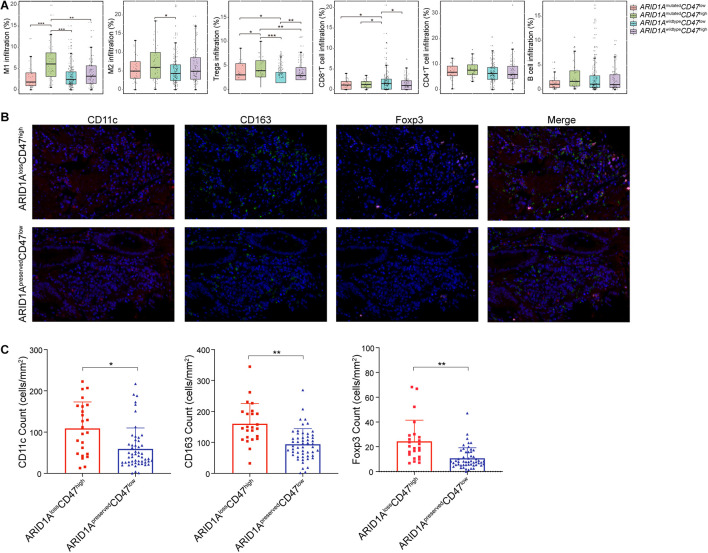
Characterization of tumor-infiltrating immune cells based on ARID1A and CD47 expression. **(A)** Estimation of tumor-infiltrating immune cells in different subgroups stratified by *ARID1A* status and *CD47* expression. **(B)** Multiplex immunofluorescent images illustrate GC tissues with ARID1A^loss^CD47^high^ or ARID1A^preserved^CD47^low^ expression that express CD11c, CD163, and Foxp3. **(C)** Quantitative analysis of the densities of immune markers between ARID1A^loss^CD47^high^ and ARID1A^preserved^CD47^low^ GC tissues. Error bars represent mean with SD. *p < 0.05, **p < 0.01, ***p < 0.001.

To understand the survival difference in different subgroups stratified by CD47 expression and ARID1A status, we further investigated the tumor-infiltrating immune cell infiltration difference in different subgroups stratified by low/high *CD47* expression and mutated/wild-type *ARID1A* expression by using quanTIseq and EPIC algorithms in the analysis of the data from TCGA. As shown in Figure 5A, higher proportion of M1-polarized macrophages were found in the *ARID1A*^mutated^*CD47*^high^ subgroup than any other subgroups. A previous study showed that high M1-polarized macrophages correlated with a better outcome in GC patients (Zhang et al., 2015). However, a recent study indicated that M1 macrophages have an attenuated prognostic value in *CD47*^high^ GC patients (Shi et al., 2021). Thus, the status of M1 macrophage might lose its prognostic value under high CD47 expression condition in GC. Lower proportions of immunosuppressive regulatory T cells (Tregs) were found in the *ARID1A*^wildtype^*CD47*^low^ subgroup than any other. Besides, the infiltration proportion of M2-polarized macrophages in *ARID1A*^mutated^*CD47*^high^ subgroup was significantly higher than that in the *ARID1A*^wildtype^*CD47*^low^ subgroup. In the *ARID1A*^wildtype^*CD47*^low^ subgroup, the proportion of CD8^+^ T cells was higher compared with any other subgroups. No difference was found in CD4^+^ T-cell and B-cell infiltration proportions. To further validate the immune cell infiltration implicated by the computational analyses, we carried out multispectral immunofluorescence assay to measure the infiltration of M1 macrophages, M2 macrophages, and Tregs in GC tissues with ARID1A^loss^CD47^high^ or ARID1A^preserved^CD47^low^ expression. Consistent with the computational analyses, multispectral immunofluorescence assays showed that higher proportions of M1-polarized macrophages, M2-polarized macrophages, and Tregs were found in the ARID1A^loss^CD47^high^ subgroup than in the ARID1A^preserved^CD47^low^ subgroup (Figures 5B,C). Putting side-by-side this information, we concluded that better prognosis of GC patients in ARID1A^preserved^CD47^low^ group might correlate with low Tregs and low M2 macrophages cell infiltration.

## Discussion

Because of its durable and robust responses, immune checkpoint therapy has taken oncotherapy into a new era. Although immune checkpoint therapy, including anti–PD-1/PD-L1 and anti–CTLA-4 therapy, has shown great successes in solid tumors (Hodi et al., 2010; Topalian et al., 2012), there are still limitations due to its narrow therapeutic window. CD47 is an emerging target for cancer immune checkpoint therapy by its functioning as a “Don’t eat me” signal to avoid the phagocytosis by macrophages (Logtenberg et al., 2020). Also, one study showed that CD8^+^ T cells and dendritic cells could exert antitumor effects through the blocking effect of CD47 (Liu et al., 2015). In line with these findings, we found that high expression of CD47 indicated a poorer outcome and was an independent prognostic factor for GC.

Most *ARID1A* mutations have been shown to be inactivated non-sense mutations or frameshift mutations, which result in the loss of ARID1A protein expression (Wiegand et al., 2010; Wu and Roberts, 2013; Wu et al., 2014). Thus, we investigated the loss of ARID1A expression in GC tissues. Zhai et al. (2016) found that ARID1A loss impaired the formation of ovarian cancer. Mathur et al. (2017) found that ARID1A loss impaired enhancer-mediated gene regulation and drives colon cancer in mice. In the present article, we found that loss of ARID1A expression was associated with a worse outcome for GC patients and was an independent prognostic factor. In line with our results, Kim et al. (2019) demonstrated that loss of ARID1A expression indicated an inferior outcome in GC patients regardless of OS or recurrence-free survival.

A previous study found that loss of ARID1A could promote tumors to become susceptible to an immune checkpoint inhibitor and could be used as a prognostic biomarker for immune checkpoint therapy (Shen et al., 2018). In terms of mechanism, loss of ARID1A could affect the efficacy of immunotherapy through damaging mismatch repair, promoting tumor mutation, up-regulating the expression of PD-L1 protein, and regulating the immune microenvironment (Shen et al., 2018). Kim et al. (2019) found that loss of ARID1A up-regulated the expression of PD-L1 through activating AKT signaling, and PI3K inhibition could down-regulate PD-L1 expression. In addition, loss of ARID1A could inhibit repayment of DNA single-strand breaks, which up-regulates the expression of PD-L1 (Shen et al., 2015; Sato et al., 2017). Thus, we analyzed the relationship between the promising immune checkpoint CD47 and ARID1A. The results showed that high expression of CD47 was significantly correlated with ARID1A loss/mutation. In GC cell lines, we also found a significant increase of CD47 at protein and mRNA levels after knockdown of ARID1A.

Our analysis on TCGA data showed that the infiltration proportion of M1-polarized macrophage was higher in *ARID1A*^mutated^*CD47*^high^ subgroup than in any other subgroups. Previous research indicated that M1-polarized macrophage played an antitumor role and was correlated with better prognosis in GC (Zhang et al., 2015). Nevertheless, we found that M1 infiltration failed to predict prognosis, which might be due to the fact that CD47 attenuates the anticancer impact of M1-polarized macrophage (Shi et al., 2021). In addition, one should bear in mind that the prognostic value of immune cells is influenced by many factors, such as cell subpopulation, spatial distribution, cell type, and functional status (Bruni et al., 2020). Also, our finding might be indicative of the loss of function of M1-polarized macrophages in CD47 high expression condition. We also found a higher infiltration proportion of Tregs in *ARID1A*^mutated^*CD47*^high^ expression subgroup than in any other groups, and a higher proportion of CD8^+^ T cells were infiltrated in *ARID1A*^wildtype^*CD47*^low^ expression subgroup than in any other one. Multispectral immunofluorescence assay has validated the infiltration proportion of M1 macrophages, M2 macrophages, and Tregs in GC tissues with ARID1A^loss^CD47^high^ or ARID1A^preserved^CD47^low^ expression. A previous study reported that CD8^+^ T cells were associated with a good prognosis, and Tregs predicted a poor prognosis (Bruni et al., 2020).

Evidently, it is of value to investigate ARID1A and CD47 expression in GC. Our study is the first to explore the relationship between loss of ARID1A expression and high CD47 expression in GC. Nevertheless, our study bears some limitations, including a not very large sample size, which necessitate conducting further investigations along this line of research.

## Conclusion

In conclusion, we showed for the first time that loss or mutation of ARID1A was significantly correlated with high CD47 expression in GC. Knockdown of ARID1A significantly increased the expression of CD47 in GC cell lines. With respect to the underlying mechanism in play, we found that ARID1A could bind to the promoter region of *CD47* to regulate its expression. Moreover, we found that ARID1A^loss^CD47^high^ expression was indicative of a poor prognosis in GC patients, and combination of ARID1A and CD47 was identified to be a promising prognosis factor in GC.

## Data Availability Statement

The original contributions presented in the study are included in the article/Supplementary Material, further inquiries can be directed to the corresponding author/s.

## Ethics Statement

Written informed consents were provided by this company and the research was conducted in accordance with recognized ethical guidelines. The studies involving human participants were reviewed and approved by the Ethics Committee of Shanghai Jiao Tong University School of Medicine Affiliated Ruijin Hospital. The patients provided written informed consent to participate in this study.

## Author Contributions

CY and JZ: conception and design. QZ, QC, SY, JJ, and ZZ: development of methodology, acquisition of data, and analysis and interpretation of data. QZ and JZ: writing — review of the manuscript. QZ, CY, and JZ: study supervision. All authors contributed to the article and approved the submitted version.

## Conflict of Interest

The authors declare that the research was conducted in the absence of any commercial or financial relationships that could be construed as a potential conflict of interest.

## Publisher’s Note

All claims expressed in this article are solely those of the authors and do not necessarily represent those of their affiliated organizations, or those of the publisher, the editors and the reviewers. Any product that may be evaluated in this article, or claim that may be made by its manufacturer, is not guaranteed or endorsed by the publisher.

## Footnotes

1
https://portal.gdc.cancer.gov/

2
http://www.cbioportal.org/

3
http://icbi.at/quantiseq

4
http://epic.gfellerlab.org
