# Supplementary material for: Combinatorial Analysis of AT-Rich Interaction Domain 1A and CD47 in Gastric Cancer Patients Reveals Markers of Prognosis
Source: Front Cell Dev Biol. 2021 Nov 3;9:745120. doi: 10.3389/fcell.2021.745120 (PMC8595398; doi:10.3389/fcell.2021.745120)
Supplement: Supplementary file 1 [file Data_Sheet_1.docx]

**Table S1. The sequences of shRNA Target**

| Identifier | Forward(5’-3’) |
| --- | --- |
| *shARID1A#1* | GCCTGATCTATCTGGTTCAAT |
| *shARID1A#2* | CCGTTGATGAACTCATTGGTT |
