# Supplementary figures and images for: Combinatorial Analysis of AT-Rich Interaction Domain 1A and CD47 in Gastric Cancer Patients Reveals Markers of Prognosis
Source: Front Cell Dev Biol. 2021 Nov 3;9:745120. doi: 10.3389/fcell.2021.745120 (PMC8595398; doi:10.3389/fcell.2021.745120)

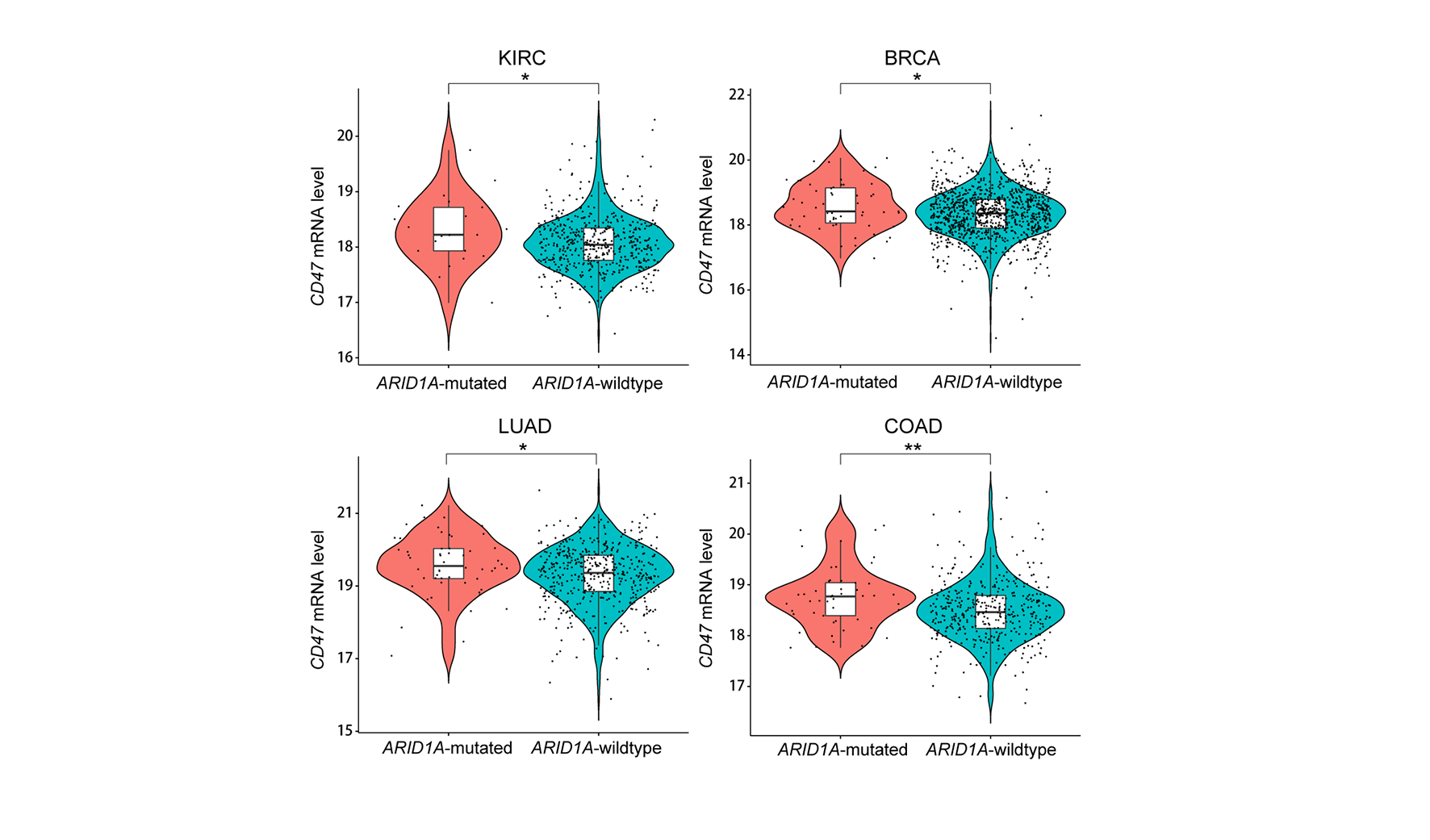

Supplement: Supplementary file 2 [file Image_1.tif]
