# Supplementary material for: Combinatorial Analysis of AT-Rich Interaction Domain 1A and CD47 in Gastric Cancer Patients Reveals Markers of Prognosis
Source: Front Cell Dev Biol. 2021 Nov 3;9:745120. doi: 10.3389/fcell.2021.745120 (PMC8595398; doi:10.3389/fcell.2021.745120)
Supplement: Supplementary file 3 [file Presentation_1.pdf]

Supplementary Figure 1

Relationship between mutated/wildtype expression of *ARID1A* and *CD47* expression in other types of cancer (data from TCGA). KIRC: kidney renal clear cell carcinoma, BRCA: breast invasive carcinoma, LUAD: lung adenocarcinoma, COAD: colon adenocarcinoma. *$p<0.05$, **$p<0.01$.
